# Allelic Heterogeneity and Trade-Off Shape Natural Variation for Response to Soil Micronutrient

**DOI:** 10.1371/journal.pgen.1002814

**Published:** 2012-07-12

**Authors:** Seifollah Poormohammad Kiani, Charlotte Trontin, Matthew Andreatta, Matthieu Simon, Thierry Robert, David E. Salt, Olivier Loudet

**Affiliations:** 1 INRA, UMR1318, Institut Jean-Pierre Bourgin, Versailles, France; 2 Department of Horticulture and Landscape Architecture, Purdue University, West Lafayette, Indiana United States of America; 3 Laboratoire d'Ecologie, Systématique, et Evolution, Université Paris-Sud XI, Orsay, France; University of Georgia, United States of America

## Abstract

As sessile organisms, plants have to cope with diverse environmental constraints that may vary through time and space, eventually leading to changes in the phenotype of populations through fixation of adaptive genetic variation. To fully comprehend the mechanisms of evolution and make sense of the extensive genotypic diversity currently revealed by new sequencing technologies, we are challenged with identifying the molecular basis of such adaptive variation. Here, we have identified a new variant of a molybdenum (Mo) transporter, MOT1, which is causal for fitness changes under artificial conditions of both Mo-deficiency and Mo-toxicity and in which allelic variation among West-Asian populations is strictly correlated with the concentration of available Mo in native soils. In addition, this association is accompanied at different scales with patterns of polymorphisms that are not consistent with neutral evolution and show signs of diversifying selection. Resolving such a case of allelic heterogeneity helps explain species-wide phenotypic variation for Mo homeostasis and potentially reveals trade-off effects, a finding still rarely linked to fitness.

## Author Summary

Plants are studied for their ability to adapt to their environment and especially to the physical constraints to which they are subjected. It is expected that they evolve in promoting genetic variants favorable under their native conditions, which could lead to negative consequences in other conditions. One approach to study the mechanisms and dynamics of these adaptations is to discover genetic variants that control potentially adaptive traits, and to study directly these variants in wild populations to try to reveal their evolutionary trajectory. We have identified a new polymorphism in a gene coding for a transporter of molybdenum (an essential micronutrient for the plant) in Arabidopsis; we show that this variant has strong phenotypic consequences at the level of plant growth and reproductive value in specific conditions, and that it explains a lot of the species diversity for these traits. Especially, the variant is associated with a clear negative effect under molybdenum-deficient conditions (caused by soil acidity) and with a subtle positive effect under molybdenum-plethoric conditions. Interestingly, the landscape distribution of the variant is not random among Asian populations and correlates well with the availability of molybdenum in the soil at the precise location where the plants are growing in the wild.

## Introduction

Some of the most important constraints that plants have to adapt to are those related to soil properties [1], [2]. These are also possibly some of the least well studied constraints, because they are spatially heterogeneous, thus not prone to typical geographic clines [3], [4], and require analysis at the local/population scale [5]. In this context, quantitative genetics approaches hold great promise to reveal the genetic basis of adaptation by enabling the identification of the molecular origin of phenotypic differences between populations or even between species [6], [7]. One of the benefits of identifying the causative polymorphism(s) and/or gene(s) explaining natural phenotypic variation is that it allows direct testing for correlations between environmental factors populations may be responding to and the occurrence of the target genetic polymorphism. This contrasts with working indirectly through populations phenotype, which may reflect contradictory patterns and trade-offs, if not genetic drift [5], [8]. Moreover, this approach also enables direct testing for fitness advantages or potential cost of adaptation (i.e. antagonistic pleiotropy, an expected argument for local adaptation), that may be masked by linkage to deleterious mutations or genetic drift in reciprocal transplant experiments [9], [10]. Molecularly identified examples of potentially-adaptive variation are still largely lacking and the debate is open as to the scale and rate of adaptive evolution [11], [12].

In this work, we aimed at identifying the molecular bases of natural variation in accumulation of an essential micronutrient and understanding the ecological significance of this diversity. We describe both the fitness trade-offs of this variation and its potential adaptive advantage in the environment, revealing a system that is unlikely to have remained neutral.

## Results/Discussion

**Figure 1:**
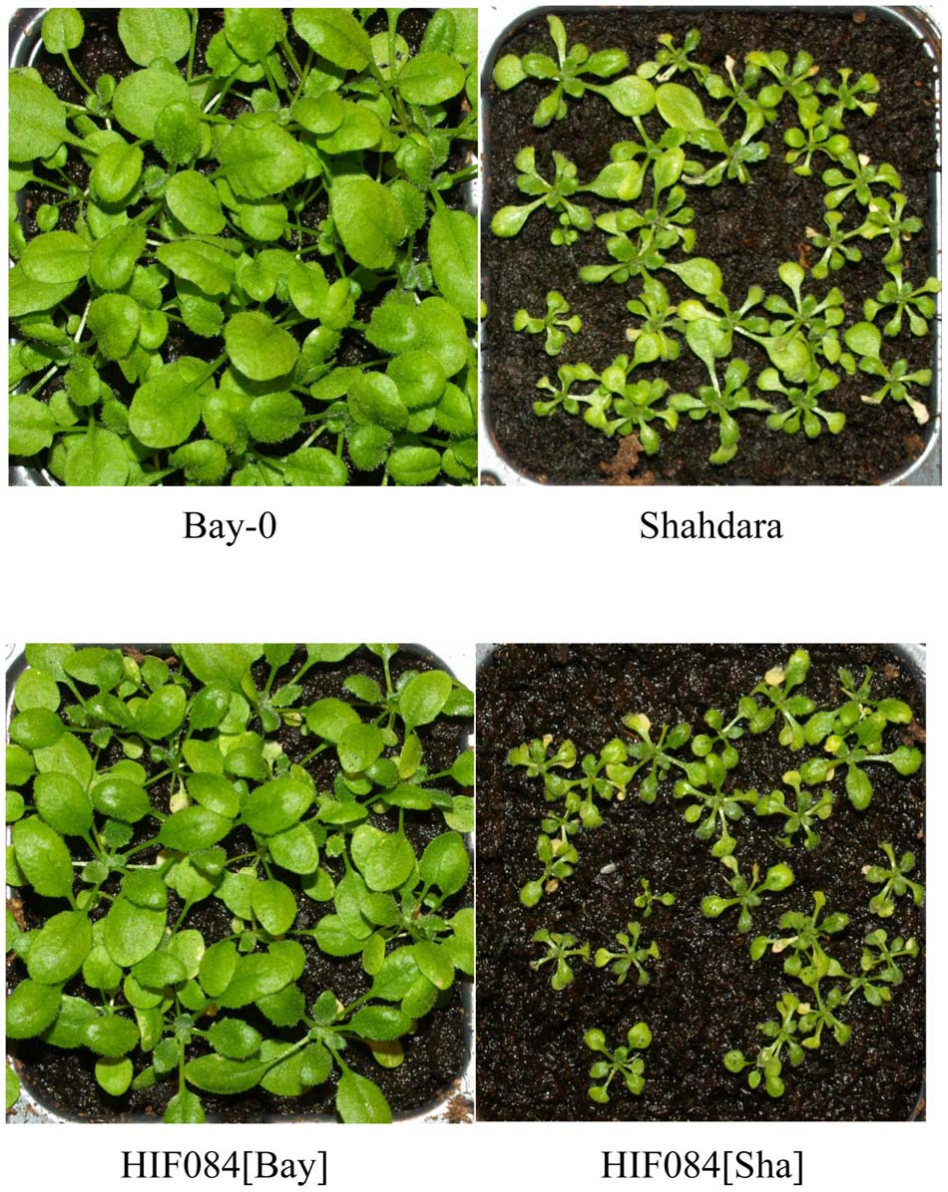
Acidic peatmoss substrate induces severe growth defect linked to the Shahdara allele. When grown on peatmoss at a pH close to 5, Shahdara is subject to severe growth and developmental arrest, necrosis and death, contrary to Bay-0 which develops normally. In the cross between these two strains, this phenotype is entirely controlled by a single locus (Figure S1), as confirmed by near-isogenic line ‘HIF084’ segregating solely for a region of chromosome 2.

**Figure 2:**
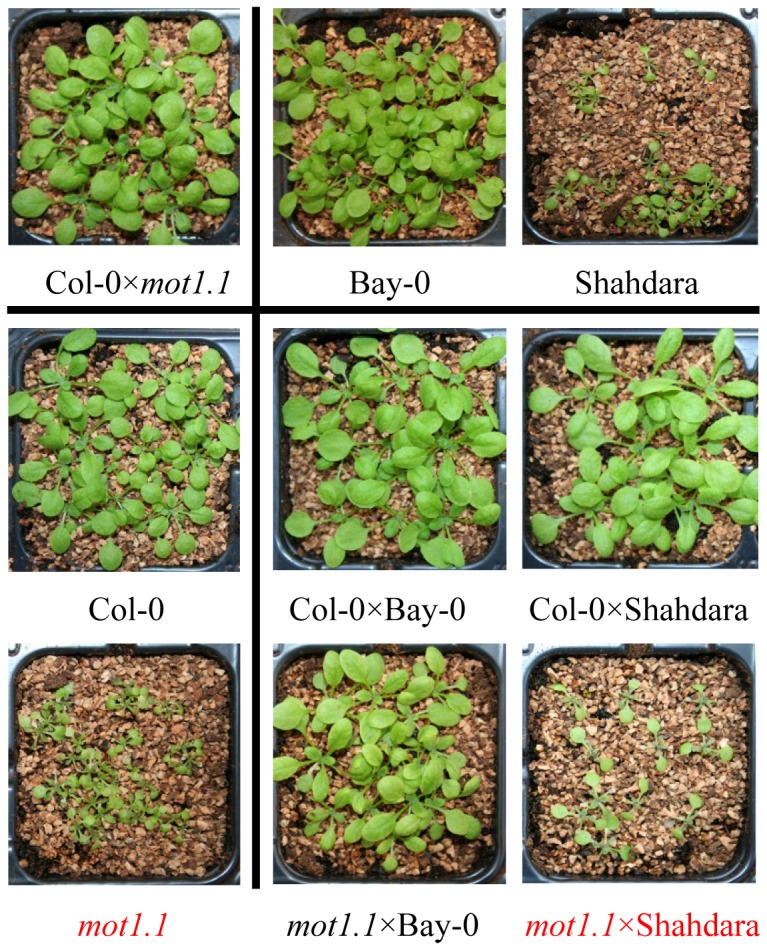
Mutant analysis and allelic complementation confirms *MOT1* as the likely causative gene. Peatmoss phenotype of diverse genotypes is shown, including Bay-0 and Shahdara parental lines, *mot1.1* mutant and its wild-type genetic background (Col-0), and F1 plants from complementation crosses between these genotypes. Unlike other alleles, the Shahdara allele at *MOT1* is not able to rescue the mutant phenotype.

**Figure 3:**
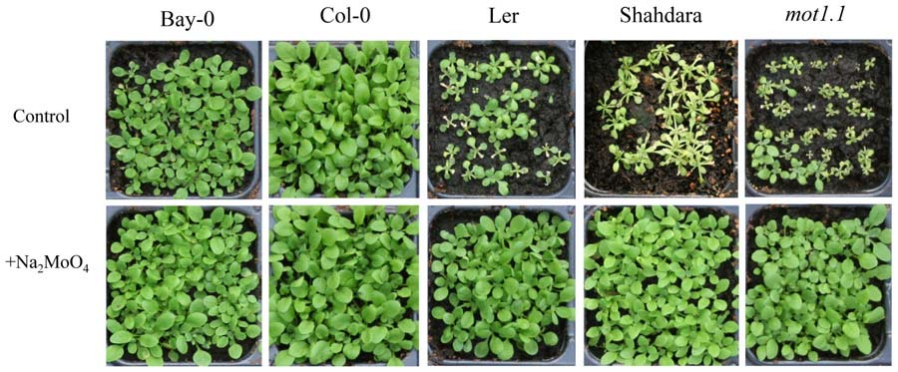
Chemical complementation links growth defect with Mo shortage. Accessions with potentially functional (Bay-0, Col-0) and defective *MOT1* alleles (L*er*, Shahdara, *mot1.1*) were grown on peatmoss substrate watered with nutrient solution containing either traces of Mo (‘Control’) as in Figure 1, or 1 mM Na_2_MoO_4_ (‘+Na_2_MoO_4_’). pH was checked to remain unchanged across treatments at ∼5.

Bay-0 and Shahdara, two strains (accessions) derived from wild populations of *Arabidopsis thaliana*, show contrasted growth behavior when grown on acidic peatmoss substrate (Figure 1). Using a segregating population derived from the cross of these two accessions, we determined the Shahdara growth defect to be segregating from a major-effect recessive locus on chromosome 2, as confirmed by a near-isogenic line derived from a residual heterozygous interval in one of the recombinant inbred lines (HIF084; Figure 1). Additional recombinant lines were phenotyped and genotyped to pinpoint the causative interval to 80 kb (Figure S1), covering 19 annotated genes. Among these, one gene appeared as a good functional candidate: *MOT1* was previously linked to the transport and homeostasis of the essential micronutrient molybdenum (Mo) in the plant [13], [14], an element which availability is known to vary with soil pH [15]. Indeed, a T-DNA insertion mutant in the *MOT1* gene (*mot1.1*; Figure S1) shows a phenotype similar to Shahdara on peatmoss and –contrary to the Bay and Col alleles– the Sha allele is not able to restore wild-type growth when combined with the mutant allele in F1 hybrids (Figure 2). Moreover, we show that defective growth is complemented by either increasing soil pH with additional CaCO_3_ mixed to the peatmoss (Figure S2) or increasing Mo availability (without altering soil pH) by adding Mo in the watering solution (Figure 3). Hence, genetic and chemical complementation shows that acidic soil pH is responsible for reducing Mo-bioavailability and that, combined with a defective allele at *MOT1*, this results in the typical Mo-deficiency syndromes of reduced leaf Mo contents, strongly altered growth and development, necrosis [16]. These observations of a significant phenotypic consequence of variation at *MOT1* provide a model for the potential adaptive significance of this variation that goes beyond the simple variation in Mo content revealed previously [13], [14].

Although we find that Landsberg *erecta* (L*er*) has a similar behaviour than Shahdara in our conditions (Figure 3), this defective allele (*MOT1* ^L*er*^) used initially to reveal the gene's activity [13], [14] is functionally different from the *MOT1* ^Sha^ defective allele. *MOT1* ^Sha^ doesn't bear the promoter 53 bp-deletion as in L*er* (Figure S1) and in fact is not showing *MOT1* ^L*er*^-like transcriptional down-regulation compared to *MOT1* ^Bay^ or *MOT1* ^Col^ (Figure S3). Instead, *MOT1* ^Sha^ seems defined by a single amino-acid change in the protein relative to Bay-0 and Col-0 (Figure S1), strongly suggesting that MOT1^Sha^ is hypofunctional. However, the MOT1 protein produced from the Sha allele is still able to increase Mo accumulation when heterologously expressed in yeast (Figure S4).

We then genotyped a random worldwide sample of ∼300 accessions for the Sha-like amino-acid change and the L*er*-like 53-bp deletion and find that these alleles are both present at intermediate frequencies (15–20%) among the populations. Sequencing 102 of these accessions for the whole gene and promoter region revealed that the very conserved *MOT1* ^Sha^ haplotype is indeed clearly defined solely by the D104Y amino-acid change, while the *MOT1* ^L*er*^ genotype is more complex and diverse (Table S1). All Sha-like and L*er*-like accessions that have been phenotyped show that both *MOT1* ^Sha^ and *MOT1* ^L*er*^ haplotypes are perfectly associated with defective growth under acidic soil conditions (Table S1) and complementation crosses with five additional Sha-like accessions confirm allelism to *mot1.1* (Figure S5). Taking into account this allelic heterogeneity now explains most of the species variation toward low-Mo contents revealed in previous work [13] (http://www.ionomicshub.org/arabidopsis/). This form of complexity –in addition to genetic heterogeneity– is probably more frequent than previously thought in many organisms and is likely to help explain part of the missing heritability [17], [18].

Regarding *MOT1* defective haplotypes, *MOT1* ^Sha^ is confined to ‘West-Asia’ (including Russia) with a high frequency among these populations (Figure S6) and displays a very low polymorphism level (πSha = 0,00016) in comparison to other haplotype clusters, including the worldwide-distributed *MOT1* ^Ler^ allele (πL*er* = 0,0017; Table S1). This may translate a recent and rapid expansion of the Sha allele through ‘West-Asia’, which could be due to neutral processes such as gene surfing associated with post-glaciation recolonization events from Central Asia [19]. This may also witness local positive selection events in favour of the Sha allele. Indeed, patterns of nucleotide polymorphisms at *MOT1* in the sample of 102 accessions strongly deviate from the expectation under the strict neutrality model, contrarily to two control loci, *PI* and *COI1* (Table S2). Negative values of Tajima's D reveal an excess of rare alleles at *MOT1*, suggesting the possible occurrence of at least one past selective sweep that has targeted this locus. Other well documented evolutionary processes such as population expansion after the last glaciation event [20] and population genetic structure [21] could also have contributed to the excess of rare alleles observed at the genome-wide level [22], as well as at the *MOT1* locus in *A. thaliana*. Nevertheless, the HKA and McDonald-Kreitman tests, which do not rely on the frequency spectrum, support the hypothesis of diversifying selection at the species level (Tables S2 and S3). The excess of within-species polymorphisms relatively to inter-specific divergence and the excess of non-synonymous polymorphisms observed at *MOT1* may result from the selection of different haplotypes at the worldwide scale. Interestingly, this trend is also clear when considering only accessions from ‘West Asia’, suggesting that the selection process could happen at different geographical scales.

**Figure 4:**
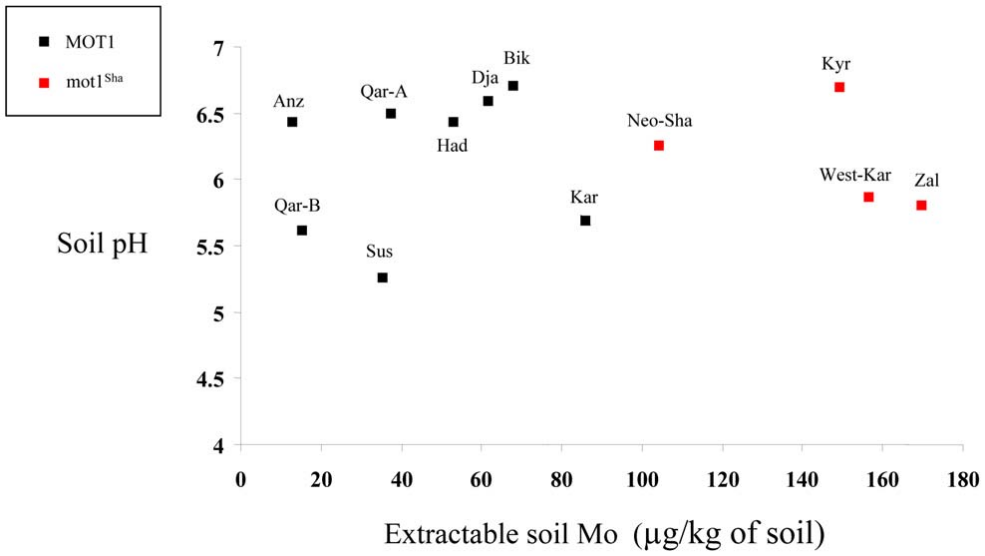
West-Asian populations highlight the correlation between *MOT1* ^Sha^ allele and high native soil Mo content. Soil parameters (pH, extractable Mo) from the precise original collection site are represented for independent populations carrying (red dots) or not (black dots) the *MOT1* ^Sha^ allele.

Our own documented collection of wild populations from diverse regions in ‘West-Asia’ allowed us to investigate potential relationships between *MOT1* alleles and environmental parameters described precisely at the population site, especially soil properties. We saw no relationship with soil pH (indeed, none of the described populations were facing acidic soil conditions), but there was an obvious trend for populations with the defective *MOT1* ^Sha^ allele to grow on soils with high water-extractable Mo content (Figure 4). This may indicate that the defective Sha allele is a protective response to Mo accumulation in environments with excess Mo. Indeed, under such conditions in the laboratory, we observe a strong decrease in fitness (through the total number of seeds produced per plant) in all genotypes (Figure S7), indicating that plants have to find the right balance between Mo deficiency and Mo toxicity, a trade-off that could be resolved partly through variation in function of the Mo transporter MOT1. Moreover, we show that a defective *MOT1* allele (either *mot1.1* or *MOT1* ^Sha^) is accompanied by a slightly increased average seed mass (another component of fitness) specifically under Mo-toxic conditions (Figure S7), the outcome of which is difficult to estimate in nature [23]. It is however worth noting that previous studies in *A. thaliana* have shown positive effects of increased seed size for example on subsequent root and shoot growth [24] or seedling survival under limiting conditions [25].

In summary, we have identified a new functional variant at *MOT1* that contributes to explain most of the species' diversity in Mo homeostasis, and associated phenotypes that provide likely explanations for its non neutral evolution and its correlation to native soil. It is still a rare finding to be able to relate functional genetic variants to fitness or trade-off effects [26]–[28], and even more to associate this variation to the environment [3], [7]. Our work indicates that environmental parameters of importance, such as soil properties, may be heterogeneously distributed and therefore require local description [5] and study of local adaptation [9], [11], which is greatly facilitated by the identification of the causative locus.

## Materials and Methods

All accessions used and the Bay-0×Shahdara RIL set [29] were obtained from Versailles Arabidopsis Stock Centre (http://dbsgap.versailles.inra.fr/vnat/). Heterogeneous Inbred Family ‘HIF084’ was derived from RIL084 (segregating for the region of interest) as previously described [30]. New collections of *A. thaliana* accessions were partly described previously [31] and are shown at http://www.inra.fr/vast/collections.htm. T-DNA insertion mutant *mot1.1* corresponds to line SALK_118311 as described [13], [14]. Genetic complementation tests were performed on F1 plants issued from the cross of diverse accessions to *mot1.1* or its wild-type background.

Acidic soil assays were performed on ‘Floratorf’ peatmoss (Floragard, Germany) mixed with CaCO_3_ (4 g per liter of dry peatmoss) to maintain a soil pH∼5, watered with classical nutrient solution and grown under typical long-day conditions at 20°C. Chemical complementations were achieved in the same condition but, either with 8 g CaCO_3_ per liter of peatmoss to reach a pH∼6, or using a watering solution supplemented with 1 mM Na_2_MoO_4_. Mo toxicity was tested on regular fertilised soil mix (pH = 6) watered with nutrient solution supplemented with 7 mM Na_2_MoO_4_, or not (control).

*MOT1* sequencing, qPCR analysis of expression (normalised against *GAPDH* and *PP2A*), functional characterisation in yeast were performed as previously described [13]. Extractable Mo was determined in soils by the method of Soltanpour and Schwab [32] using ICP-MS as the detector.

A total of 102 *A. thaliana* accessions (including 48 accessions known to maximize *A. thaliana* diversity [33] and 44 accessions from ‘West-Asia’) and 5 *A. halleri* accessions (I-14, I-16, F-1, PL-22 and TZC; obtained from H. Frérot at Univ. Lille [34]) were sequenced at *MOT1* (including 1 kb upstream and 0.3 kb downstream for *A. thaliana* accessions) and at two reference loci, *COI1* (At2g39940; 2,600 bp coding sequence) and *PI* (At5g20240; 2,150 bp coding sequence). Those genes were either used previously as reference or shown to have a neutral pattern of polymorphisms in *A. thaliana* [35], [36]. Sequences were aligned using Codoncode Aligner v3.7.1. and subsequent alignments were improved visually.

Intraspecific analyses *i.e.* nucleotide diversity estimated by π [37] and θ_w_ [38], and Tajima's D statistics [39] were calculated using DNAsp v5.10.01 on the whole region sequenced. Ten thousands coalescent simulations under the strict Wright-Fisher neutral model assuming no recombination and conditioning on S were performed to estimate statistical significance of Tajima's D.

For interspecific analyses, the orthologous of *MOT1* and of the two control loci in *A. halleri* were used. The McDonald-Kreitman test [40] was performed by using DNAsp v5.10.01 in order to test for possible excess or deficiency in replacement substitutions at *MOT1*. Singletons were discarded for this analysis in order to reduce the contribution of slightly deleterious mutations (expected at very low frequencies and unlikely to become fixed). Neutral index was calculated as previously described [41]. Divergence between *A. thaliana* and *A. halleri*, defined as the average number of nucleotide differences between populations per gene, was calculated using DNAsp v5.10.01 and used to perform HKA tests with the multilocus HKA program available from J. Hey laboratory (http://genfaculty.rutgers.edu/hey/software).

## Supporting Information

Figure S1 Fine-mapping the causative locus identifies *MOT1* as a candidate gene. A. The physical region of chromosome 2 found to be linked to the growth defect phenotype is shown (physical positions are given in Mb). B. Zooming in the candidate region highlights recombinants within the inbred lines that allow to fine-map the causative locus, thanks to additional markers (vertical dashed lines). Individual lines' genotype are depicted in horizontal coloured boxes (green = Bay allele; purple = Sha allele ; dashed = heterozygous) and their phenotype on peatmoss are indicated (S = Sensitive; R = Resistant). C. This allows to narrow down the causative region to 80 kb, a region containing 19 predicted genes including the candidate At2g25680 (*MOLYBDATE TRANSPORTER 1*). D. *MOT1* has been sequenced in parental accessions and polymorphisms between Col-0, Bay-0 and Shahdara are represented along the single-exon gene model, including an amino-acid change specific to Shahdara (D104Y). The position of the insertion of a T-DNA in the *mot1.1* mutant (SALK_118311) is indicated.
(TIF)

Figure S2 Chemical complementation links growth defect with soil pH. Increasing soil pH from ∼5 (‘Control’) to ∼6 (‘+CaCO_3_’) by doubling the amount of CaCO_3_ mixed to the peatmoss substrate rescues normal vegetative growth of the Shahdara strain.
(TIF)

Figure S3 *MOT1* transcript accumulation does not explain *MOT1* ^Sha^ defective allele. *MOT1* transcript accumulation relative to *GAPDH* and *PP2A* controls is shown from roots of diverse genotypes as in Figure 3. Contrary to L*er* and *mot1.1*, Shahdara accumulates normal levels of transcript. Standard errors are shown.
(TIF)

Figure S4 MOT1^Sha^ is able to transport Mo in yeast. The Sha allele of MOT1 was overexpressed in yeast heterologous system (Sha/p416) and shown to lead to Mo accumulation compared to the empty vector (p416, used as reference) or the yeast wild-type strain (BY4741), to an extent not significantly different from the Col MOT1 allele (Col/p416). Error bars represent interquantile range of medians.
(TIF)

Figure S5 Multiple accessions sharing the *MOT1* ^Sha^ haplotype confirm the causative gene and polymorphism. Peatmoss phenotype of diverse genotypes is shown: 5 independent Sha-like accessions (Stw-0, Kly-2, Sij-4, Kondara and Zal-3) and F1 plants from complementation crosses between each of these accessions and either the *mot1.1* mutant or its wild-type genetic background (Col-0). As in Figure 2, all accessions sharing the *MOT1* ^Sha^ haplotype are both sensitive and unable to rescue the mutant phenotype.
(TIF)

Figure S6 Worldwide distribution of functionally contrasted alleles at *MOT1*. Original collection site and functional *MOT1* haplotype (Sha-like in red dots, L*er*-like in yellow, Col-like in blue) is shown on a world map for the 102 accessions sequenced in Table S1. L*er*-like accessions are found across the whole species known distribution range, while Sha-like accessions are restricted to Asia and Russia (‘West-Asia’).
(TIF)

Figure S7 Effect of Mo toxicity on fitness components -seed number and weight- of contrasted *MOT1* genotypes. The fitness consequences of Mo toxicity was tested on regular (non-acidic) soil mix with different nutrient solutions containing either traces of Mo (‘Control’) or 7 mM Na_2_MoO_4_ (‘Na_2_MoO_4_ (7 mM)’). The assay was performed to compare (A) the *mot1.1* mutant and its wild-type (‘WT’) genetic background, (B) the Bay and Sha allele in the HIF084 background (‘HIF[Bay]’ vs ‘HIF[Sha]’). In both cases, the defective *MOT1* allele is represented with black bars. To avoid heterogeneity/effects on descendance conveyed through the maternal plant, the mutant assays were performed as a progeny testing from a mother plant segregating for the T-DNA insertion. Two fitness parameters are represented: the total number of seeds produced per plant (on the left) and the weight of 1,000 seeds (on the right). Error bars show 95% confidence interval of the mean. For the weight of 1,000 seeds, there is no significant difference between genotypes under ‘control’ treatment, while defective *MOT1* alleles have significantly larger seeds under Mo excess (t-test; p<0.017 when comparing *mot1.1* and WT; p<0.0018 when comparing HIF084[Bay] and HIF084[Sha]).
(TIF)

Table S1 Haplotype diversity at MOT1 among 102 accessions. Polymorphisms detected across 102 *A. thaliana* accessions for the *MOT1* locus, including 1 kb upstream (promoter region) and 0.3 kb downstream of the coding region (between blue vertical double-lines). The position (coordinates) of polymorphic bases (regions) are indicated in bp from TAIR10 reference. Synonymous polymorphisms are highlighted in light grey, non-synonymous polymorphisms in medium grey and missing data in dark grey. *A. lyrata* and *A. halleri* serve as outgroups. Accessions individually phenotyped on acidic peatmoss substrate are indicated (S = Sensitive; R = Resistant). The main functional haplotypes highlighted with colours (Sha-like in red, L*er*-like in yellow, Col-like in blue) are those represented on Figure S6, including the Sha-like haplotype defined by the ‘D104Y’ polymorphism, and the Ler-like haplotype associated to the “53 bp-deletion.”
(XLS)

Table S2 Genetic diversity and tests of selection at *MOT1* and two reference loci (*COI1* and *PI*).
(XLS)

Table S3 Detailed results of HKA simulations.
(XLS)
