# Supplementary figures and images for: Allelic Heterogeneity and Trade-Off Shape Natural Variation for Response to Soil Micronutrient
Source: PLoS Genet. 2012 Jul 12;8(7):e1002814. doi: 10.1371/journal.pgen.1002814 (PMC3395621; doi:10.1371/journal.pgen.1002814)

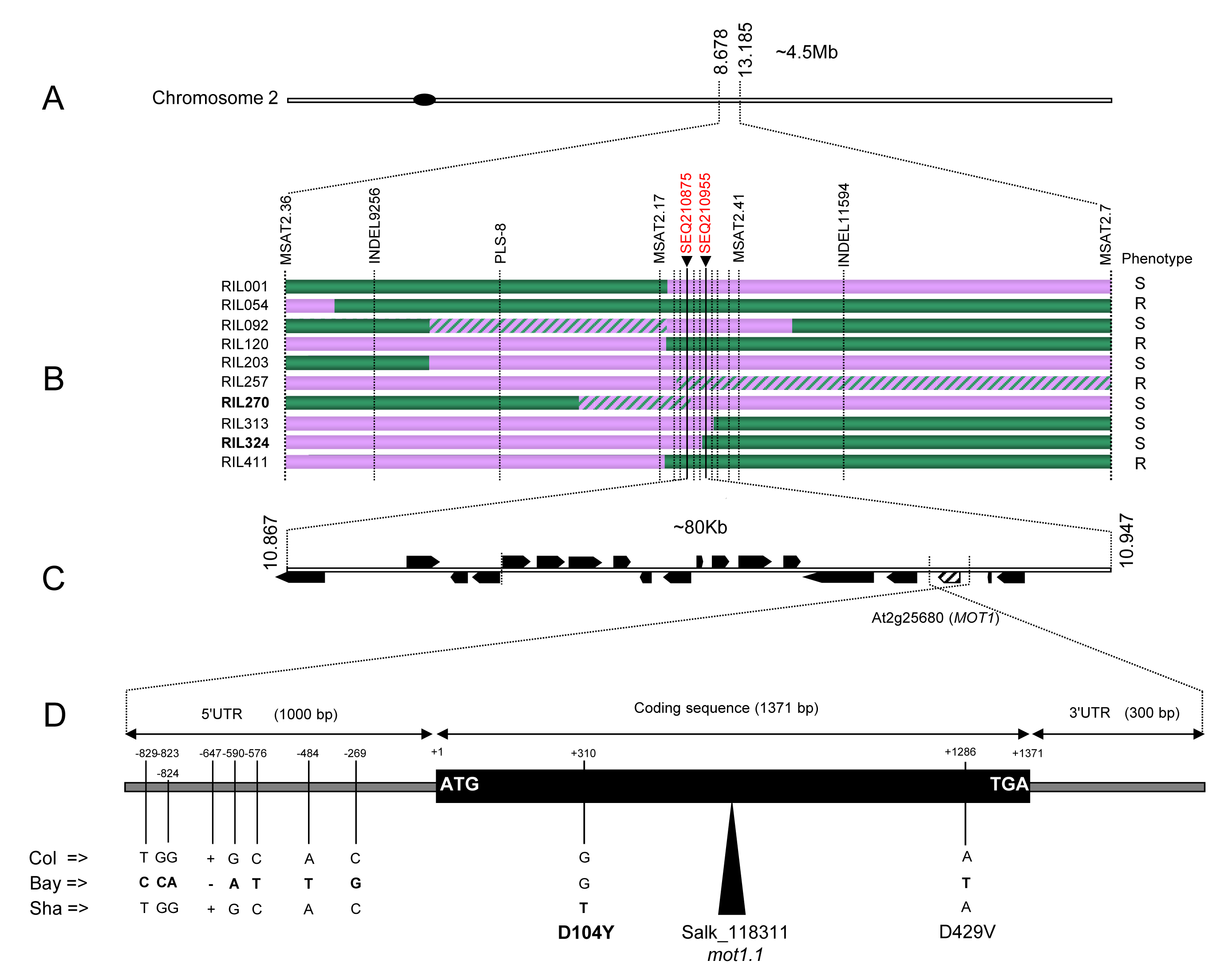

Supplement: Figure S1 — Fine-mapping the causative locus identifies MOT1 as a candidate gene. A. The physical region of chromosome 2 found to be linked to the growth defect phenotype is shown (physical positions are given in Mb). B. Zooming in the candidate region highlights recombinants within the inbred lines that allow to fine-map the causative locus, thanks to additional markers (vertical dashed lines). Individual lines' genotype are depicted in horizontal coloured boxes (green = Bay allele; purple = Sha allele ; dashed = heterozygous) and their phenotype on peatmoss are indicated (S = Sensitive; R = Resistant). C. This allows to narrow down the causative region to 80 kb, a region containing 19 predicted genes including the candidate At2g25680 (MOLYBDATE TRANSPORTER 1). D. MOT1 has been sequenced in parental accessions and polymorphisms between Col-0, Bay-0 and Shahdara are represented along the single-exon gene model, including an amino-acid change specific to Shahdara (D104Y). The position of the insertion of a T-DNA in the mot1.1 mutant (SALK_118311) is indicated. (TIF) [file pgen.1002814.s001.tif]

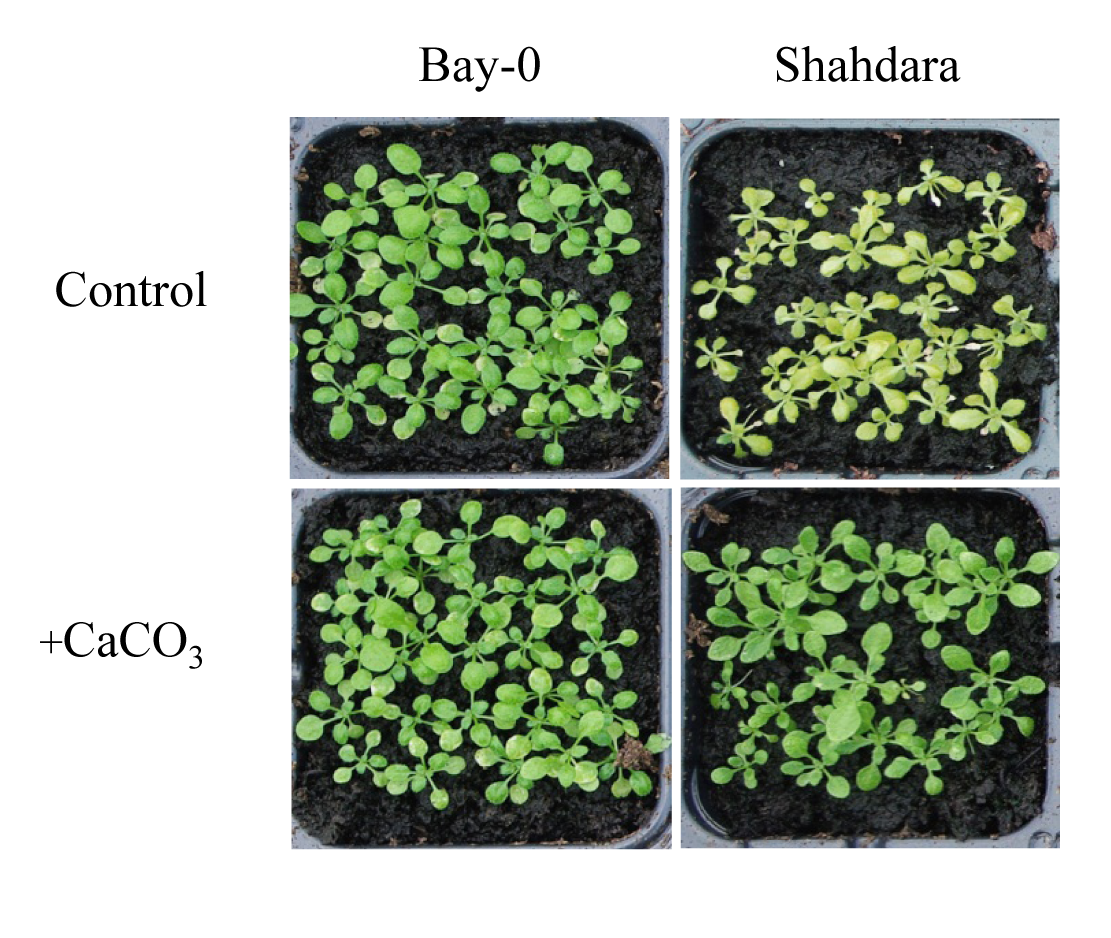

Supplement: Figure S2 — Chemical complementation links growth defect with soil pH. Increasing soil pH from ∼5 (‘Control’) to ∼6 (‘+CaCO3’) by doubling the amount of CaCO3 mixed to the peatmoss substrate rescues normal vegetative growth of the Shahdara strain. (TIF) [file pgen.1002814.s002.tif]

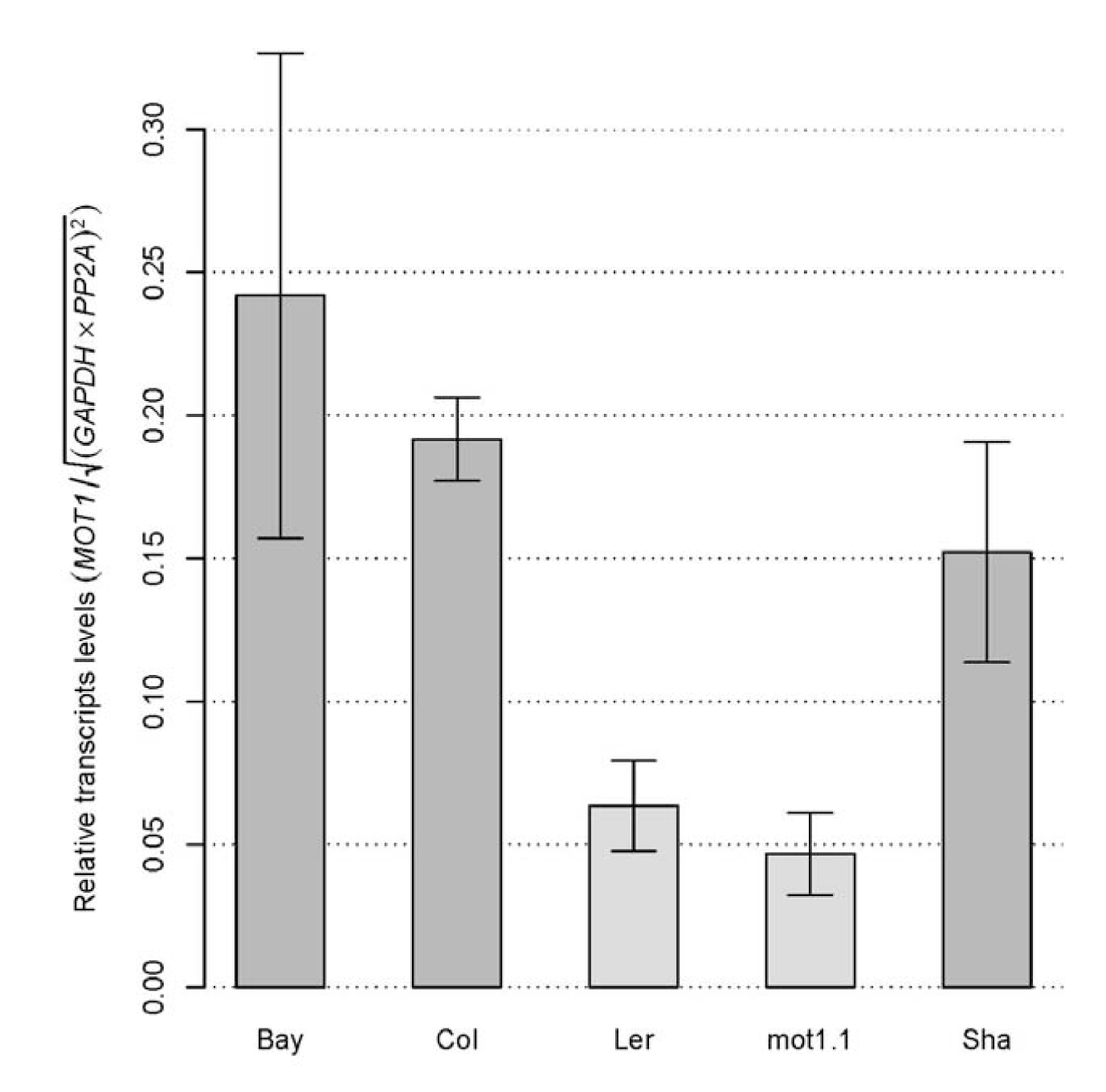

Supplement: Figure S3 — MOT1 transcript accumulation does not explain MOT1 Sha defective allele. MOT1 transcript accumulation relative to GAPDH and PP2A controls is shown from roots of diverse genotypes as in Figure 3. Contrary to Ler and mot1.1, Shahdara accumulates normal levels of transcript. Standard errors are shown. (TIF) [file pgen.1002814.s003.tif]

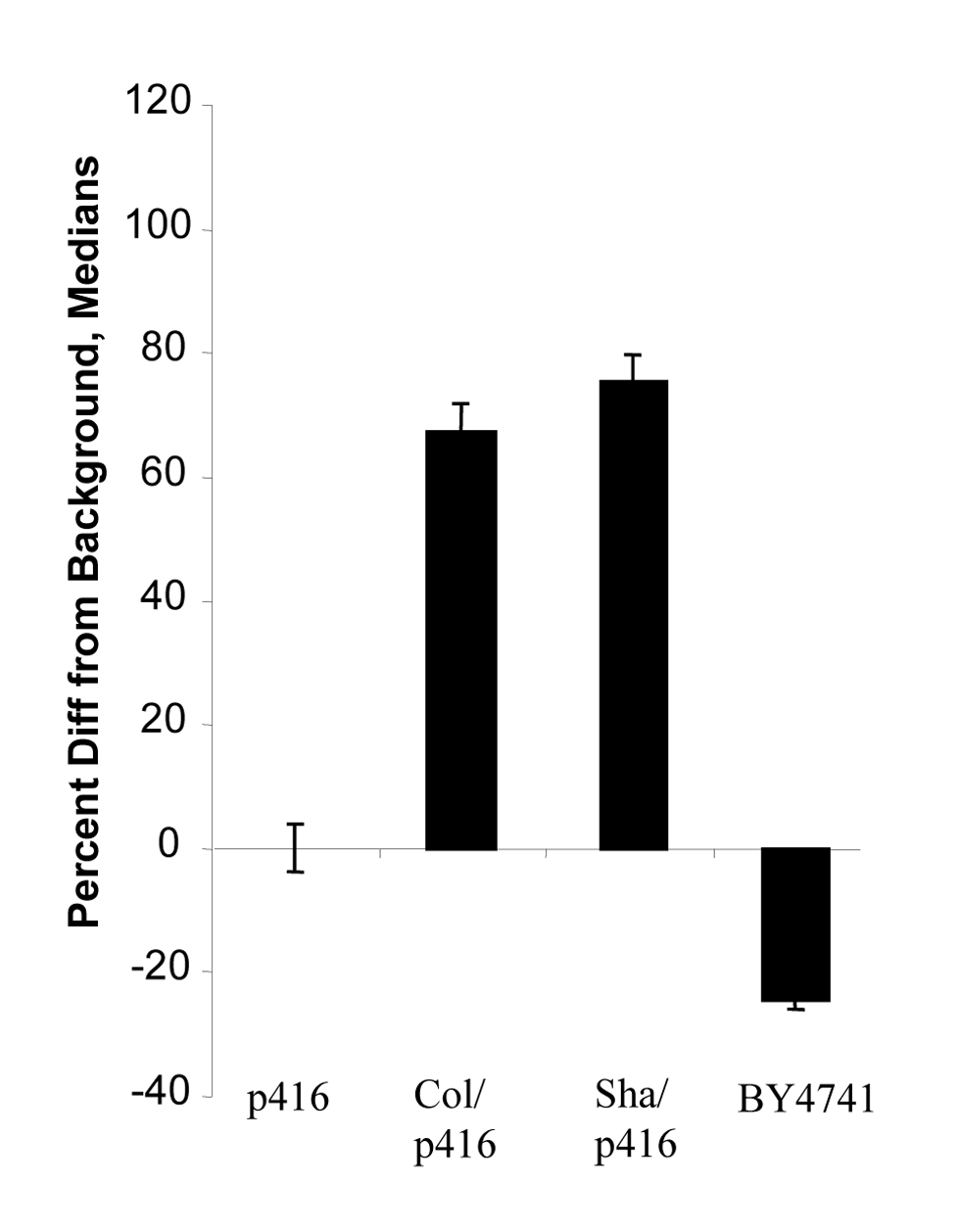

Supplement: Figure S4 — MOT1Sha is able to transport Mo in yeast. The Sha allele of MOT1 was overexpressed in yeast heterologous system (Sha/p416) and shown to lead to Mo accumulation compared to the empty vector (p416, used as reference) or the yeast wild-type strain (BY4741), to an extent not significantly different from the Col MOT1 allele (Col/p416). Error bars represent interquantile range of medians. (TIF) [file pgen.1002814.s004.tif]

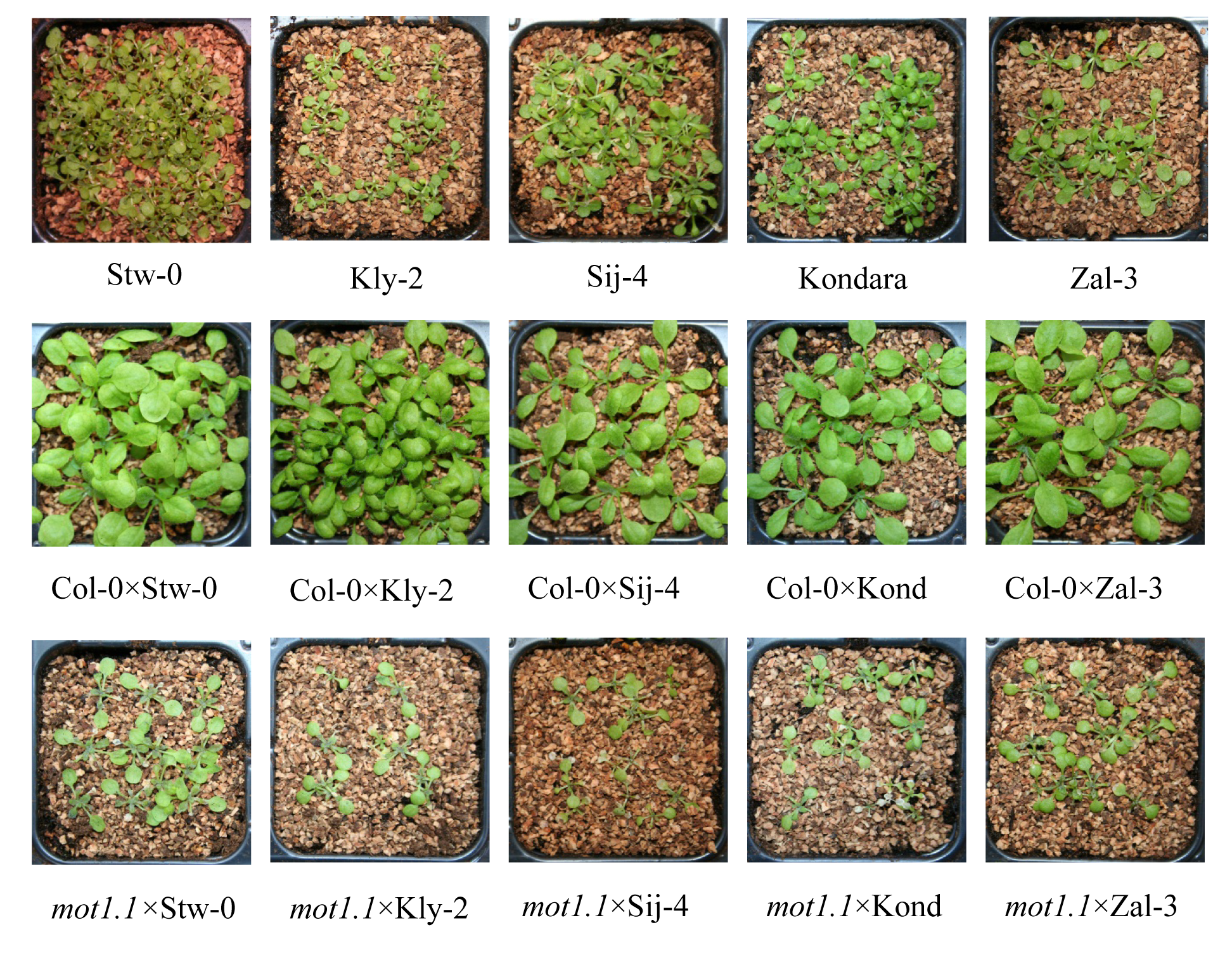

Supplement: Figure S5 — Multiple accessions sharing the MOT1 Sha haplotype confirm the causative gene and polymorphism. Peatmoss phenotype of diverse genotypes is shown: 5 independent Sha-like accessions (Stw-0, Kly-2, Sij-4, Kondara and Zal-3) and F1 plants from complementation crosses between each of these accessions and either the mot1.1 mutant or its wild-type genetic background (Col-0). As in Figure 2, all accessions sharing the MOT1 Sha haplotype are both sensitive and unable to rescue the mutant phenotype. (TIF) [file pgen.1002814.s005.tif]

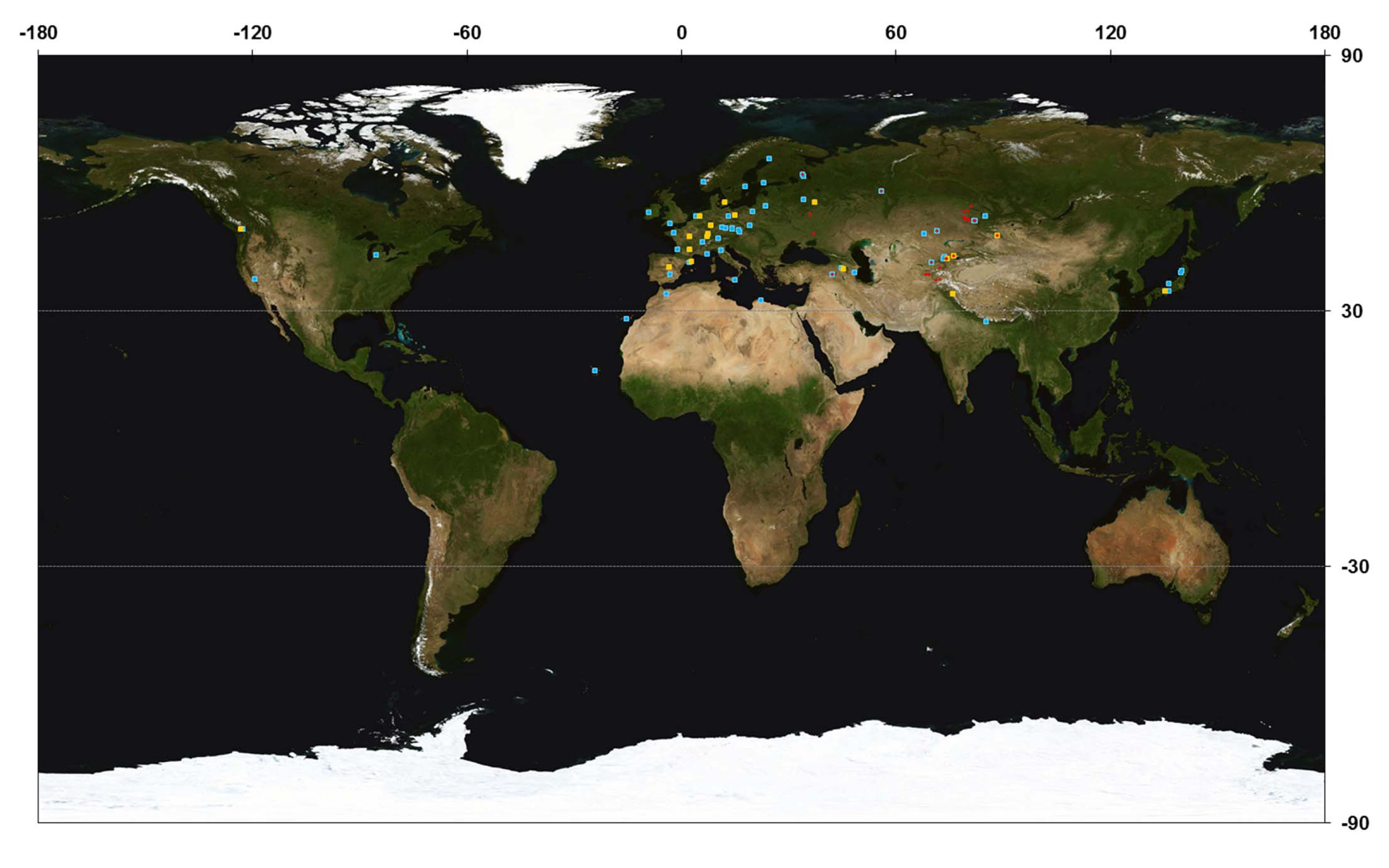

Supplement: Figure S6 — Worldwide distribution of functionally contrasted alleles at MOT1. Original collection site and functional MOT1 haplotype (Sha-like in red dots, Ler-like in yellow, Col-like in blue) is shown on a world map for the 102 accessions sequenced in Table S1. Ler-like accessions are found across the whole species known distribution range, while Sha-like accessions are restricted to Asia and Russia (‘West-Asia’). (TIF) [file pgen.1002814.s006.tif]

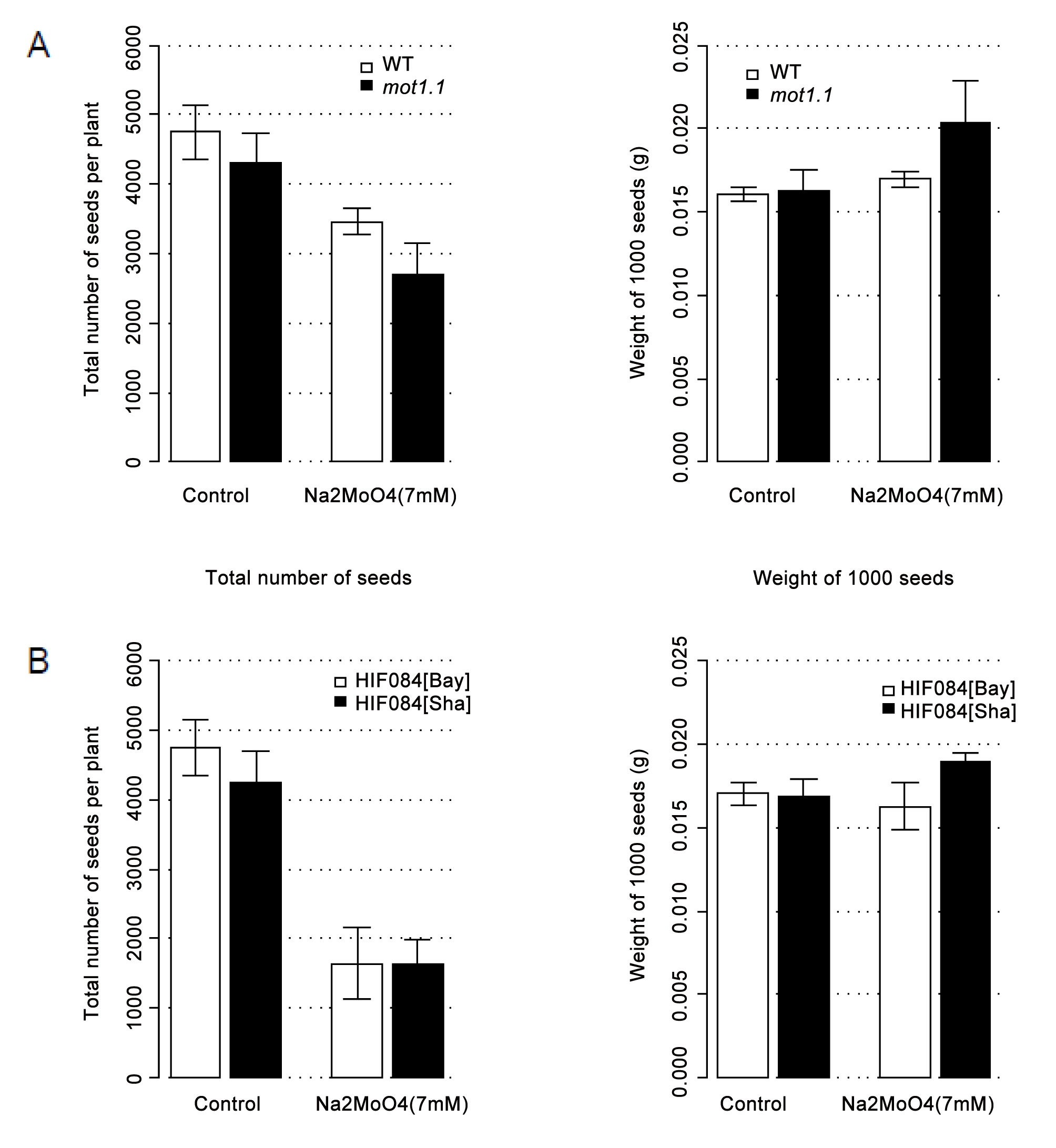

Supplement: Figure S7 — Effect of Mo toxicity on fitness components -seed number and weight- of contrasted MOT1 genotypes. The fitness consequences of Mo toxicity was tested on regular (non-acidic) soil mix with different nutrient solutions containing either traces of Mo (‘Control’) or 7 mM Na2MoO4 (‘Na2MoO4 (7 mM)’). The assay was performed to compare (A) the mot1.1 mutant and its wild-type (‘WT’) genetic background, (B) the Bay and Sha allele in the HIF084 background (‘HIF[Bay]’ vs ‘HIF[Sha]’). In both cases, the defective MOT1 allele is represented with black bars. To avoid heterogeneity/effects on descendance conveyed through the maternal plant, the mutant assays were performed as a progeny testing from a mother plant segregating for the T-DNA insertion. Two fitness parameters are represented: the total number of seeds produced per plant (on the left) and the weight of 1,000 seeds (on the right). Error bars show 95% confidence interval of the mean. For the weight of 1,000 seeds, there is no significant difference between genotypes under ‘control’ treatment, while defective MOT1 alleles have significantly larger seeds under Mo excess (t-test; p<0.017 when comparing mot1.1 and WT; p<0.0018 when comparing HIF084[Bay] and HIF084[Sha]). (TIF) [file pgen.1002814.s007.tif]
